# News Items Concerning Third Pan-American Medical Congress

**Published:** 1901-01

**Authors:** 


### News Items Concerning Third Pan-American Medical Congress.

Dr. H. A. West, of Galveston, a member of the Executive Committee and Chairman of the Section of Medical Jurisprudence, Pan-American Medical Congress, sends us the following:

The Organization Committee has postponed the meeting of the Third Pan-American Medical Congress until Feb. 4th. It will be held from that date until the 8th inclusive. All literature pertaining to the Congress is in Spanish, but occasional news items will be sent to the weekly journals.

Delegates can go either by the land routes, which are all via Florida, or by the water route from New York. Full particulars concerning these routes, except that of the Ward Line steamer from New York, will be found in the A. M. A. Journal of Nov. 3d. Those coming from south of Washington and west of Pittsburg will probably select the Florida route, while those from the northeast will find the Ward Line more convenient. The steamer Seguranza of the Ward Line leaves New York Jan. 30th, arriving in Cuba on Feb. 3d, the day before the beginning of the Congress. The new Ward Line steamer, Morro Castle, holding 135 cabin passengers, leaves Havana on Feb. 9th, reaching New York on Feb. 11th. The round trip, everything included, is $60.00. Anyone going via this route will be absent from New York twelve days.

Any of the delegates going down by the Ward Line, who desire it, may by a supplementary payment of $25.00 return via Santiago.

This trip will take from ten to twelve days, during which time the passengers live on the ship, and have their meals there if they desire. The party goes from Havana by rail to Cienfuegos in one afternoon, there taking the steamer to Santiago, and from Santiago to Nassau in the Bahamas, and from Nassau to New York. The stay in these different ports will be for a day or more.

English speaking members of the Cuban Reception Committee will meet all steamers on their arrival in Havana and escort the delegates to the hotels assigned to them. The best hotels in Havana are Telegrafo, Mascotte, Inglaterra and Pasaje. Rates $3 to $5 a day.

The Entertainment Committee, appointed by the City Council of Havana, for the purpose of making it pleasant for the delegates during their stay, have arranged a program, which, although as yet not determined upon in every detail, is nevertheless one which adds an attractive feature to the meeting.

There will be a large ball given at the Tacon theatre. This will be under the management of a Sub-Committee and an auxiliary Ladies' Committee. The committee is sparing no pains to make the ball the great social feature of the week, and one that will be most enjoyable, not only to the delegates, but also to any ladies in their parties. (There will be a banquet given at the same place). On another day an excursion will be made to the sugar plantation of Mr. LaCoste near Havana on three government transports. On arriving there the delegates will be shown the manner of growing sugar cane, gathering it, grinding, cooking and refining the sugar derived therefrom, as well as all other points of interest connected with the method of making sugar and life on a sugar estate.

Refreshments will then be served either on the estate or on the transports returning. These transports will be so arranged for the guests as to give the greatest amount of comfort and pleasure during the trip. On another day there will be a parade of the police and an exhibition of the fire companies, in which they will show the promptness and skill with which they combat any fires taking place in the city. Receptions will also be given by various officials and private dinners by numerous Cuban physicians to the delegates with whom they are acquainted.

The three general sessions of the Congress, at which papers of general interest will be presented, and addresses delivered by official representatives from the different countries, will be held at the Tacon theatre in Havana. The section meetings will be held in the University on the first floor, and on the first and second floors of the Institute. The delegates from Mexico are at present in the lead, and a large number of abstracts have been received from them.

